# A Virtual Reality Platform for Analyzing Remote Archaeological Sites

**DOI:** 10.1093/iwc/iwz011

**Published:** 2019-04-16

**Authors:** Brendan Cassidy, Gavin Sim, David Wayne Robinson, Devlin Gandy

**Affiliations:** 1 Department of Computing, University of Central Lancashire, Preston PR1 2HE, UK; 2 Department of Archaeology, University of Central Lancashire, Preston PR1 2HE, UK; 3 Department of Archaeology, University of Cambridge, Cambridge CB2 3DZ, UK

**Keywords:** virtual reality, VR, mixed reality, archaeology, heritage, data visualization Handling Editor, Dr. SI Editors

## Abstract

This paper describes a Virtual Reality (VR) prototype developed to help archaeologists and other stakeholders explore and analyse archaeological data in a more immersive context. We describe a VR reconstruction of Pleito Cave, a fragile world class rock-art site with accessibility limitation. Key stakeholders are identified and a prototype is described that provides a VR platform for visualizing and interacting with complex archaeological data (gathered from techniques such as decorrelation stretch and X-ray fluorescence) virtually ‘in ***situ’***, in a way that would not be possible at the real site. The prototype allows multiple remote users to interact with the cave together remotely providing opportunities for collaborative interpretation and analysis of archaeological data. We also present a survey-based evaluation in which both archaeologists and Native American stakeholders indicate positive responses for measures of both engagement and value.

## 1 Introduction

Archaeologists commonly use reality capture techniques such as Photogrammetry and laser scanning to capture, and keep a record of archaeological sites and the artefacts found in them (Barsanti *et al*., 2015). Since the release of Virtual Reality (VR) headsets at consumer level they have become an attractive tool for museums and heritage organizations to help engage the public with historic environments and artefacts found in them. With the release of lower cost standalone VR headsets, such as Oculus Go, they are also becoming more accessible to the general public at home. While the literature on designing for virtual museums is extensive, what is less clear is to what extent VR platforms can be used not only to engage the public, but also as a research communication tool for archaeologists wanting to visualize and interact with real- world archaeological data in an immersive way. This paper describes the initial development of a platform designed to allow key stakeholders to interact with archaeological data, in addition to 3D recreations of the site. Specifically, the work is focussed on providing a platform for multiple stakeholders with archaeologists, land owners and people with a cultural connection to the sites being the main focus.

Often, important archaeological sites can be difficult to access, which restricts the stakeholder’s ability to visit the site when desired. This could be for a number of reasons: the fragility of the archaeological remains; existence on private property; the remoteness; difficulty of access; and dangers associated with natural phenomena (high cliffs, deep caves, underwater, etc.) or cultural reasons (areas of conflict, contamination, damaged buildings, etc.). Health issues such as restricted mobility, age or even transportation may equally affect the ability of individuals to experience archaeological sites. The introduction of immersive technologies into the archaeological and heritage sector presents an opportunity to overcome these access problems in new ways, and for multiple stakeholders.

**Figure 1:**
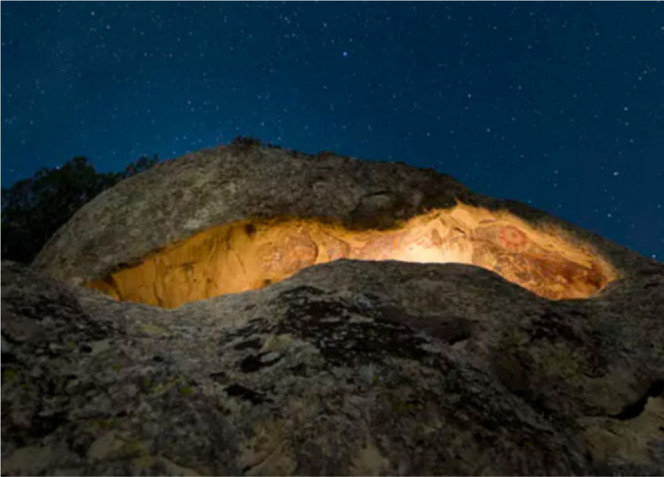
Pleito cave site.

The work presented in this paper describes an interactive recreation of Pleito cave (Fig. 1), one of the most elaborately painted rock-art sites in the world, located in the San Emigdio Mountains, California. The site has accessibility issues due to the fragile nature of the rock art found at the site and its remote geographical location. Recent work at the site has generated a lot of rich archaeological data (Bedford *et al*., 2018; Kotoula *et al*., 2018; Robinson *et al*., 2015) and makes it uniquely appropriate for prototyping immersive platforms to address issues of accessibility and archaeological data visualization. The prototype addresses accessibility issues by supporting simultaneous multi-user access and initially supports data visualization by overlaying digitally enhanced/processed textures of the rock art onto the original geometry, allowing users to walk around and interact with the processed textures *‘in situ’* to provide a viewing context previously unavailable to researchers. in effect, this is providing an ‘Enhanced Reality’ to allow archaeologists to view and interact with archaeological data not visible in a standard photo realistic reconstruction of the site.

## 2 Background and Related Work

Designing virtual heritage spaces is a well-researched area (Bekele *et al*., 2018). While the majority of virtual museum exhibits are solitary experiences for the user, Li and Zhou (2016) describe a multi-user virtual exhibit. The work describes a re-creation of an aircraft carrier using popular 3D modelling tools. While there is novelty in facilitating a multiuser experience, the models used are not taken from real data, so would have limited value where high accuracy, realistic representations of the environment are required and would not be of practical use to researchers. Christou *et al*. (2006) took a mixed approach when developing a VR system for an immersive projected CAVE style environment, where photographs and ground plans of an archaeological site were used as the basis for hand modelling a reconstruction. While this gives a more accurate general layout, it still does not produce an accurate reconstruction as there is still a reliance on artistic ability.

The work of Fleury *et al.* (2012) investigated the use of collaborative remote manipulation of 3D data for scientific analysis in a room scale virtual environment. They found that as tasks became more difficult a collaborative approach to data manipulation became more efficient than single user manipulation. This does indicate that there could be potential in developing immersive techniques to explore scientific data (in this case, relating to archaeology and heritage) collaboratively. 3D data visualization and tangible 3D interaction was used in the design of the ArcheoTUI system (*Reuter et al*., 2010), a system designed to assist with the virtual reassembly of archaeological artefacts. While this was not implemented using a VR headset, it does show the potential that immersive technologies can have when working with 3D archaeological data.

Research evaluating the impact of digital additions to artwork in a VR museum setting (Hurst *et al.* 2016) evaluated whether adding visual augmentations to a piece of art, or adding small 3D animations related to its content, had a positive impact on the experience and on how the art is perceived. The additions were still creative in nature and so are of limited use where scientific accuracy is required, but there is a suggestion from this work that manipulating artwork in a VR environment could lead to improved user experience. This view is also supported by Sim *et al*. (2018) who explored the enhancement of children’s museum experiences through the design of augmented reality within the context of a Roman museum. There is also work that suggests that VR has the potential to capture cultural and historical heritage with indigenous populations. Trescak *et al*. (2017) used a VR simulation to capture aboriginal heritage and users of the system were observed spending a lot of time in a central ‘ camp site’ area observing ‘day to day’ activities such as tool making, painting and food preparation.

Where scientific accuracy is important photogrammetry is a popular technique used to digitize archaeological sites and is well suited to cave sites large enough to allow multiple images from a camera to be taken at many different angles. The subsequent 3D models generated are then accessible on a wide range of devices, for example, Yip Chan *et al*. (2013) used tablets to render a cave in a physical space. The reconstruction was realistic, so the captured data would be of use to researchers wishing to study the site further. However, with a requirement to use a tablet as the ‘window’ into the virtual world interactions with the world are less immersive, less natural, and is unsuitable for distributed multi user scenarios. The novelty of the work in this paper is that it combines much that has been covered in the related work described and integrates a multi-user platform that is suitable for a range of target users and uses. Importantly, it can also be used as a data visualization tool for archaeologists and as a tool for Native American populations to explore their heritage.

## 3 Prototype Design

**Figure 2:**
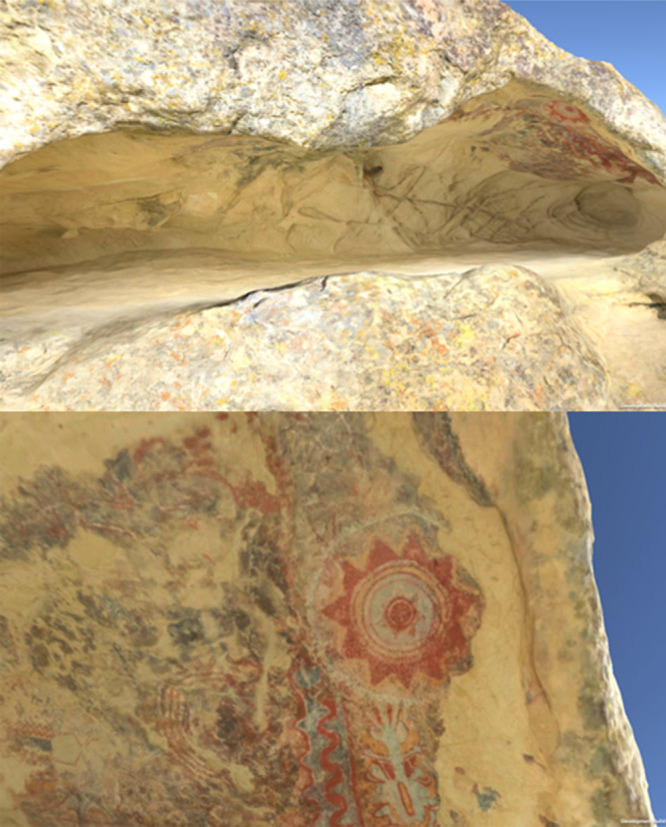
Virtual cave reconstruction and rock-art detail.

A photogrammetrically generated mesh and texture of the cave site and rock art was produced as illustrated in Fig. 2.

**Figure 3:**
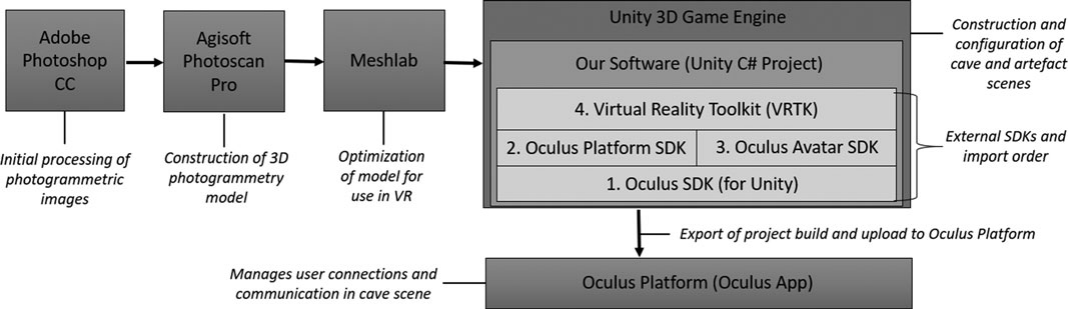
Overall pipeline/architecture of the VR platform.

The photogrammetry model of Pleito was constructed from 896 images taken with a Nikon D810 and a Nikon AF-S FX 20mmf/1.8G lens, mounted on a Manfrotto tripod. The camera was set to raw (.nef), base ISO of 64, and lens at f13. Photographs were taken at night, with the cave illuminated by five Switronix 250w TorchLED panels, each set to 4000k. Due to complex cave features, limitations of lighting placement, and brightness of LED panels, exposure varied from 2 to 15’, which allowed us to manually light paint sections of the cave if needed. We used a X-Rite Colour Checker™ incrementally throughout the session to confirm light temperature and serve as a colour correction base for post-processing. Our photogrammetry image capture protocol emphasized a > 66% overlap between images. Measurements of 20 distinctly identifiable cave features as well as eight 10 cm scales served to scale the model. Images were processed in Photoshop CC (version 18.0) and exported as JPEGs. The photogrammetry model was modelled in Agisoft PhotoScan Pro (Version 1.3.0 build 3075). The overall platform architecture can be seen in Fig. 3.

**Figure 4:**
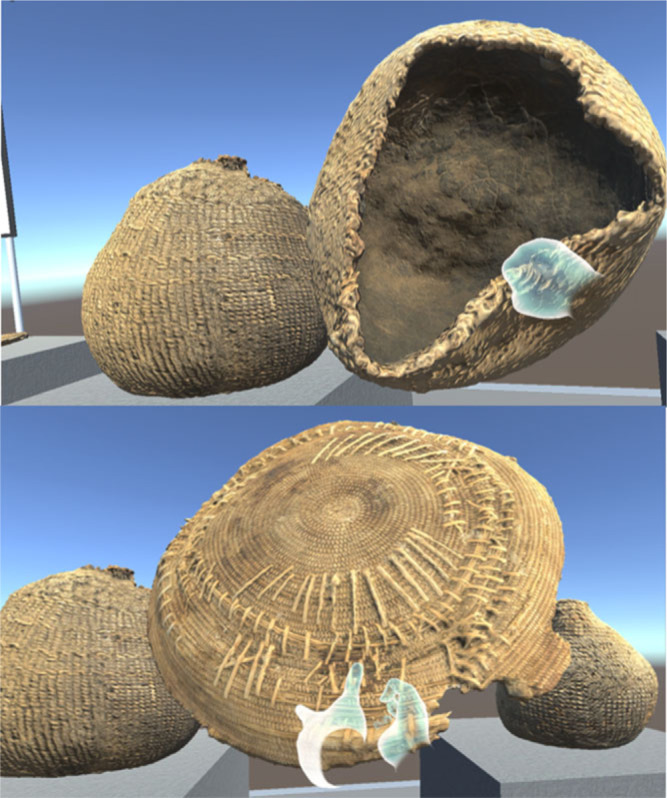
Integrated Virtual Basketry Exhibit.

Following photogrammetric generation of the cave model, it was then optimized for VR in MeshLab and imported into the Unity 3D Game engine. The Virtual Reality Toolkit software library (Ball, 2018) was used to integrate locomotion and interactivity in VR. The target device was Oculus Rift and the Oculus Avatar SDK and Oculus Touch controllers were used to provide hand presence. The Avatar SDK provides a Unity plugin supporting social features to allow colocation. This integrates directly with the Oculus platform and allows any user who is connected as a friend on the ocu- lus platform and has the prototype application installed to join them in the cave. To complement the site reconstruction, a virtual exhibition space was also developed where artefacts found at this site, or other related sites could safely be handled and past practices explored and discussed. While virtual exhibition spaces themselves are not novel (Bruno *et al*., 2010; Hurst *et al*., 2016; Wojciechowski *et al*., 2004), this is, as far as we are aware, the first time a VR artefact exhibition space has been integrated alongside an immersive archaeological data analysis & visualization tool. Tangible virtual exhibits of real artefacts that users can touch and manipulate have been popular with users in the past outside of a VR context (Figueroa *et al*., 2009). Figure 4 illustrates example basketry available to users found at related sites (see Bryne *et al*., 2016; McArthur and Robinson, 2016; Robinson, 2017).

### 3.1 Key user groups

The key stakeholders targeted with this prototype include site owners/managers, archaeologists and people who culturally identify with the site. Each user group has different needs and will want to interact with the site in a different way.

#### 3.1.1 The Tejon Indian tribe of California

The Tejon Indian tribe, a recently federally reinstated pluralistic tribe, are the key stakeholders who identify with the proposed archaeological sites. Some, if not all, of paintings found on the walls of the cave were certainly created by hands of their ancestors. An early prototype was piloted with the Tejon and other Native people to trial the immersive environment. The response was positive, with Native youths responding particularly well to the virtual environment. Equally, the prototype proved effective for use by elders who had mobility issues who could not access and navigate the site themselves. With further work, we aim to provide a platform to re-connect tribal members to sites and practices no longer in living memory.

#### 3.1.2 The Wind Wolves Preserve

The Wind Wolves Preserve are the key stakeholders who own the land where the archaeological sites are located. The Preserve’ s objective is to conserve the environment and the 150+ known archaeological sites on their property, while promoting public education. The platform is being used with visitors at their visitor centre as well as for other events. Importantly, it will be a component of their children/young people’ s education and outreach programs that form a core aspect of their mission. Furthermore, since the prototype is designed to provide access to sites not normally accessible, it is an important site management tool to deflect potential harmful visitation promoting cultural resources without damaging them.

#### 3.1.3 Archaeologists

Perhaps one of the most striking aspects of the interactive platform developed is the ability to augment real archaeological data ‘*in situ*’ onto the virtual cave walls retaining a location dependant context that is lost during the initial archaeological data collection process. This enhanced method of data visualization has promising implications for exploring, interpreting and analysing archaeological data. The remote- user support also allows collaborators and experts from around the world to immerse themselves in the prototype to bring their own expertize to exploring and interpreting the data presented.

## 4 Co-Location

**Figure 5:**
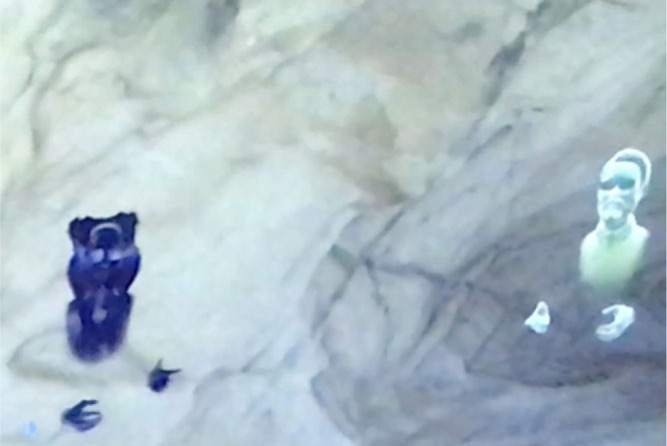
FIGURE 5. Co-location with the Avatar SDK.

One of the difficulties with inaccessible, fragile archaeological sites is that it is often difficult for key stakeholders to visit a site together. This could be because they are unable to physically get to the location of the site, or because the site itself will not support a large number of simultaneous visitors. The platform supports virtual co-location of users through use of the Oculus Avatar SDK. Fig. 5 illustrates two users colocated in the cave.

Full voice, hand and head presence is supported for up to four people allowing users to move around, discuss and point out areas of interest to each other within the cave. A remote trial of the platform was tested with domain experts at the 2018 Society for American Archaeology (*Cassidy et al*., 2018). In the trial, representatives from the three main stakeholder groups (The Wind Wolves Preserve, Tejon Indian Tribe & Archaeologists) along with the developers of the interactive platform successfully gave an informal demonstration from within the cave while simultaneously located in Washington DC, California and the UK.

## 5 Enhanced Reality

When analysing rock art, archaeologists often photograph the areas of interest and use image processing techniques such as Decorrelation Stretch (Harman, 2006) on the image to reveal or enhance detail that is difficult to see with the naked eye. By applying this technique to the photogrammetry models, high-resolution textures archaeologists are able to immerse themselves within and interact with the cave in a way they would not be able to do at the real site.

**Figure 6:**
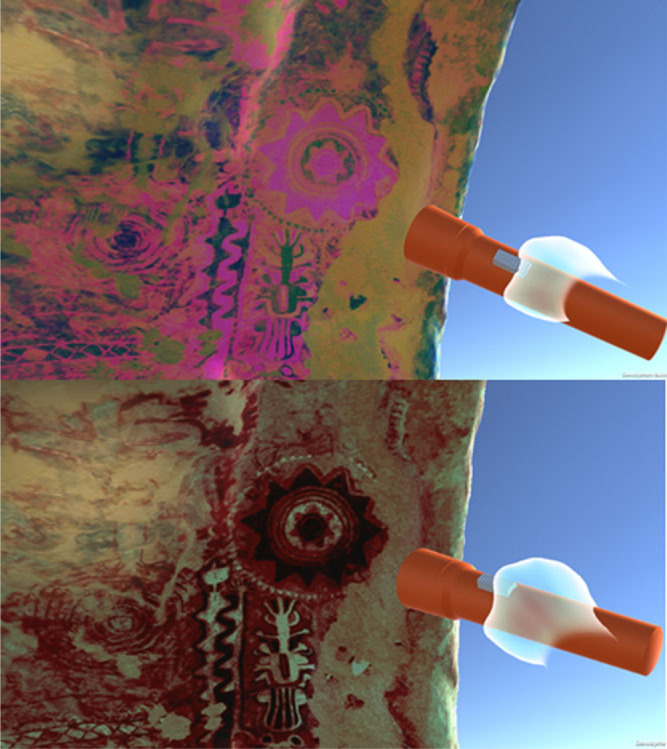
Immersive visualization of fugitive elements.

Figure 6 illustrates a ‘Flashlight’ tool within the cave that allow users to naturally explore the rock art, switching between normal and processed textures in a way that feels natural. If the user notices any unusual elements when browsing the processed textures, they are able to shine the flashlight tool to reveal the rock art in the normal colour spectrum, thus enabling a free-form tacking back-and-forth between processed and unprocessed visual data. We aim to extend this type of data visualization to support other sources of data such as paint pigment analysis.

As well as scientific analyses, rock-art analysis can also be interpretive in nature, especially when deciding what the artwork represents. Being able to provide a situational context to the different pieces of rock art to Native Americans and rock- art experts, and view them virtually *‘in situ*’ provides a level of contextual detail unavailable with traditional photographs or on-screen 3D models.

## 6 User Evaluation

As the different user groups were geographically dispersed, for this study, a survey methodology was deemed suitable to ascertain feedback on user engagement. The survey tool was e-mailed to representatives of each user group and participants who had interacted with the Pleito VR application were asked to complete a survey.

### 6.1 Participants

In total, 22 people completed and returned the survey. There was a mix of eight archaeologists, six Native Americans, four students and four general members of the public. No respondents identified themselves as staff of the Wind Wolves Preserve, though it is possible general members of the public, or students may have interacted with the VR app at the Wind Wolves Preserve visitors centre.

### 6.2 Study design

The study aimed to understand user engagement with Pleito VR, in addition it sought to determine the value this type of platform provided to the different user groups. For example, to explore ways and the extent platform can help members of the Tejon Indian Tribe to engage with their history and culture. Due to the dispersed nature of the target population, it was impossible to carry out a controlled study. The application was made available to the Tejon Indian Tribe and the Wind Wolves Preserve via a laptop and headset. The survey tool was designed and distributed through e-mail to be completed by participants during a tribal gathering where the application was available to users. There was no facilitator supervising the completion of the survey so there may be temporal differences from minutes to days from when the users interacted with the system and completed the survey.

### 6.3 Survey tool

The survey tool was designed to capture the value of the application to the end user along with a measure of user engagement and immersion. The survey was limited to one side of A4 to maximize response rate. To establish user engagement six questions were taken from the ‘short form’ version of the user engagement scale (UES) (*O’Brien et al*., 2018). In the UES survey, four subscales were identified (Reward factor, Focused attention, Perceived usability and Aesthetic appeal). To maximize response rate the appropriate two subscales ‘Reward factor’ and ‘Focused attention’ were identified and presented to users in a Likert style question format. Each subscale consisted of three statements the user was asked to agree/disagree with:

- FA: Focused Attention
  - I lost myself in this experience.
  - The time I spent using Pleito VR just slipped away.
  - I was absorbed in this experience.
- RF: Reward Factor
  - Using Pleito VR was worthwhile.
  - My experience was rewarding.
  - I felt interested in this experience.

Perceived usability and Aesthetic appeal were not evaluated, as the focus of the evaluation was on capturing the level of value the VR application contributes between user groups. The application is also still in the prototype stages, so capturing usability and aesthetic appeal data would be unreliable in this iteration of the prototype. This also ensured the survey would be a single page and allowed room for demographic data and four open-ended questions to be subject to thematic analysis. Any formative feedback about aesthetics and usability could also be captured within these open-ended questions. The questions were the following:

- What part of the Pleito VR app did you find the most valuable to you and why?
- What features do you think could be added to the Pleito VR experience to make it more useful to you for understanding the cave’ s history and why?
- What was your favourite part of the Pleito VR experience and why?
- Has the Pleito VR experience changed anything about you and how you think about the real Pleito site? (please explain how)?

The questions were designed to gain the subjective views of users while ensuring a single survey could be distributed to the different user groups.

### 6.4 Analysis

For the questions taken directly from the UES survey tool, the Likert scores were coded strongly Disagree = 1 through to Strongly agree = 7. The values for each of the two subscales (reward factor & focused attention) were calculated by calculating the mean of the three questions in each sub section. To get an overall score for engagement the mean was taken for all questions together as recommended in the original work (O’Brien *et al*., 2018), although we do acknowledge that the overall engagement score does not factor in the two missing subthemes of Perceived usability and Aesthetic Appeal.

To analyse the qualitative results, thematic analysis was used following the six phase approach reported by Nowell et al. (2017). In Phase 1 to become familiarized with the data the researchers transcribed the data into an excel spreadsheet for analysis. In phase two an initial set of codes were established by systematically going through the data and producing codes related to each statement. In Phase 3, the researchers generated themes based on the initial codes using an inductive analysis approach. Braun and Clarke (2006) define inductive analysis as a process of coding the data without trying to fit it into a pre-existing coding frame or the researcher’ s analytic preconceptions. Within the context of this research, the values of the users were being established and the researchers had no preconceived idea of what these values should be. In Phase 4, the researchers reviewed the themes to ensure they represent the original data set and modified themes accordingly. Finally one researcher examined the data and identified the core themes contained in the answers. The descriptions of the themes were then created and original answers were categorized according to the relevant themes they represented.

### 6.5 Results and discussion

The results are presented based on the Likert scale answers associated with the UES survey questions followed by the thematic analysis of the four open-ended question.

#### 6.5.1 UES survey results

Due to the relatively low response rate comparisons between user groups would not be reliable. The responses from the four different user groups who responded were aggregated for each question and results are presented in Table 1.

**Table 1:** UES survey results for ‘Focused Attention’ and ‘Reward Factor’.

Question	Mean	SD
I lost myself in this experience.	5.32	1.25
The time I spent using Pleito VR just slipped away.	4.90	1.19
I was absorbed in this experience.	6.18	0.66
Focused Attention (aggregated score)	5.28	1.18
Using Pleito VR was worthwhile.	6.36	0.84
My experience was rewarding.	6.32	0.78
I felt interested in this experience.	6.22	1.34
Reward Factor (aggregated score)	6.08	1.01

Positive scores were reported for both focused attention *(Mean (M) = 5.28, Standard Deviation* (SD) = *1.17) and reward factor subscales (M = 6.08,* SD = *1.01)* indicating the platform was valued by users.

While not directly comparable, it was noted the mean score for focused attention was lower than the score for reward factor. This may be because some of the participants may have been basing their scores on retrospection, it may have been several days or even weeks since they last used the application. Sense of reward/value could persist for longer when compared to a person’s sense of focused attention. Sense of value/reward may also grow over time as the person recalls and reflects on what they have taken away from the experience. Combining the two subscales gave an overall score for user engagement with the platform *(M =* 5.89, SD = 0.7). A Wilcoxon signed-rank test showed that there was a significant difference to the neutral value of 3.5 for overall engagement with the platform *Z* =-4.12, *p <* 0.001, *r =* -0.88. This indicates the application held overall value for users who completed the survey.

#### 6.5.2 Thematic analysis results

The results from each of the four open-ended questions were coded separately. Although some of the same themes emerged, the narrative and reasoning behind the coding differed between questions. The themes and descriptions for the first question ‘What part of the Pleito VR app did you find the most valuable to you and why?’ are shown in Table 2.

**Table 2:** Thematic analysis for the value of the app.

Theme	Description
Removing access barriers	The Pleito Cave allows people to get close and intimate with a rock-art site that is largely inaccessible to the public
Technology enhanced visualization	Benefits from examining the art with D-stretch technology; enabling you to see things that are no longer visible to the naked eye
Environment protection	Protection of the site from damage by visitors
Interaction artefacts	Examining artefacts in a risk free way
Sound effects	Sound effects increase the overall experience adding a sense of authenticity
Quality issues	Poor images detract from the experience and the VR could make users anxious
Scaling within cave	Scaling makes it more comfortable to view the artwork than in real life

There were common values across the different user groups, for example a Native American Stated ‘I liked that you could get close to and look at the painting without going there, thus not impacting the site’. A similar statement was also made by an archaeologist: ‘Ability to review rock art without disturbing the site’. The Native Americans appeared to value the interaction with the basketry with one stating ‘Picking up the basket and getting goose bumps when I did pick it up’ and another ‘to hold and look at baskets from ancestors was mind-blowing’. This demonstrates the potential of making immersive archaeological data visualization more accessible to wider stakeholders. One of the main focuses of the project was the visualization of d-stretch image processing technology within the application and this was also identified as a valuable feature by users.

The results for the second question ‘What features do you think could be added to the Pleito VR experience to make it more useful to you for better understanding the rock art or the baskets, and why?’ are presented below (Table 3).

**Table 3:** Thematic analysis for the new features.

Theme	Description
More information	More information should be provided about the artefacts such as pigments and materials used. This should be from archaeologists and Native American narrative
Enhanced landscape	The view from the cave outwards needs to be realistic, observing the rock art and spacial relationship to landscape
More layers in art	More layers to the art, for example results of XRF/Raman pigment analysis visible as a layer or the possibility to view single chronological phases more
Better sound	Having echoes in the cave to enhance the experience
Cultural information	Native language inclusion and cultural definitions

A common comment related to the lack of surrounding landscape and how that contrasted with the realistic photo- grammetrically generated cave. There was a sense of disconnection between the cave and its surrounding area. The users wanted more data available about the artefacts and the ability to provide annotations, for example ‘Pop-up explanations about design, materials (e.g. pigments, grasses), dates, cultural affiliation, etc.’ and ‘For instance, if you used a tool that clicked on the sun image at Pleito and a box came up to that showed where else that sun image occurs, like at Painted Cave in Santa Barbara.’. The suggestions related to improving the users understanding of the visuals through augmentation of additional information in either text, pictorial or audio form.

The third question examined what should be removed or modified and the results are presented in Table 4. The only thing that was suggested could be removed was an interactive bow and arrow integrated as part of the artefact exhibit scene, as this was judged to be fun but of no real value. For example, one user said ‘While the gaming (archery) part was fun for a minute, I didn’ t get a lot out of it’ . As the bow and arrow was interactive it was not photogrammetrically generated in the same way as the other artefacts, this could also highlight the importance of authenticity in such a tool, not just for archaeologists, but also for Native Americans wishing to explore past practices of their ancestors.

**Table 4:** Thematic analysis for removed or modified.

Theme	Description
Inappropriate interaction	Feel that it should be made clear that you cannot interact with artefacts like the basket in real life. There was a bow and arrow which could be interacted with and this did not really reflect the value of the app
Vertigo issue	Care needs to be taken to mitigate vertigo issues
Scale inside cave	As you are scaled you do not get a sense of the real size of the cave. You should perhaps enter in full scale before scaling is applied
Multi-modal interaction	Addition of tactile feedback to simulate the cold may improve and help emulate the real experience
Archaeology respect culture	It needs to be clear how the archaeologist respect the culture and materials
Native sounds	Add more native sounds and welcome songs

There was a range of suggestions for improving the experience including the use of multimodal interaction through tactile feedback. There was an overall sense that more could be included to make the overall experience realistic. This includes getting a sense of the overall scale of the cave as well as understanding the environmental issues such as heat and sounds.

The final question sought to ascertain whether the views of the users has changed towards the exhibited artefacts and the cave site itself, the results are shown in Table 5.

**Table 5:** Thematic analysis for changed opinion.

Theme	Description
Seeing art	Being able to see the artwork and different layers. You can see more than in the real cave, giving a heightened experience
interaction with artefacts	Could get a sense for the real life practical use of the artefacts
Preservation	Reminds of the need to preserve the site
Access and public support	Opens the site up to the public and helps raise support from the general public
Visual quality	The initial VR experience which was purely visual highlighted how important it was to consider other senses when studying an archaeological site. There needs to be high-quality graphics but the lack of sound, smells and the heat made for a different experience to the real site

There was overlap with previous discussion found in Table 2, relating to access and preservation. For example, one archaeologist stated ‘VR can bring public to fragile sites and used effectively—might increase public support.’, whilst a Native American it changed their view on this subject, they stated ‘used to feel strongly sites kept intact I felt moving or excavating would disturb the ancestors I feel it is now important for young ones to know and learn’ . The majority of users found that the experience positive and the VR capabilities enhanced the site, for example ‘I think the VR of Pleito actually demonstrates more the complexity of the art than the real cave as you can manipulate the conditions to see more’.

## 7 Conclusions and Further Work

**Figure 7:**
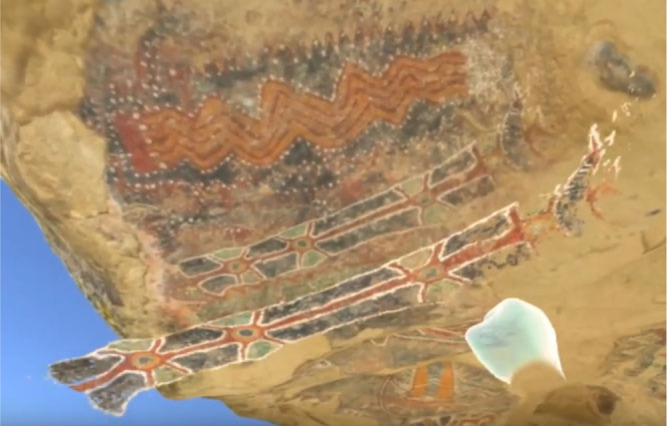
Example paint/layer separation.

Using a technique known as ‘Portable X-ray Fluorescence’ combined with advanced imaging processes, it is possible to analyse the various layers of overpainting that have occurred at the Pleito cave site (Fig. 1) over the years. Using data already gathered from the site (Bedford *et al*., 2016, 2018; Gandy and Robinson, 2018; Kotoula *et al*., 2018), we are able to reconstruct not only what the cave looks like now as a static record but also visualize how the cave would have looked over time, by starting with a virtual ‘blank canvas’ we are able to separate the layers of overpainting providing an immersive ‘walk in’ visualization of how the paint was built up over time. This unique perspective is not possible to achieve when interacting with the real site and is the next immediate focus of the work, followed by user studies aimed at identifying how stakeholders interpret the data when presented in different ways. For data visualization and analysis purposes, archaeologists and rock-art interpreters are able to separate layers of paint and even remove specific layers from the cave surface for comparison and analysis against other pieces of art in the cave. Figure 7 illustrates an early example of paint layer manipulation within the tool.

The survey results indicate the system used to visualize archaeological data within the cave has value to both archaeologists and Native American rock-art interpreters. It also indicated that authenticity is important, even for non-scientific purposes. There is an appetite to interact with and visit areas that are too fragile or remote to visit in everyday life. The ability to interact with historical artefacts directly linked to personal heritage can be profound, even eliciting physiological responses in a Native American participant (goose bumps). Archaeologists indicated an increased desire to interact with their data in an immersive way and this prototype has demonstrated the potential of immersive data visualization in this context. Further work is now needed to extend the techniques highlighted above to offer a more diverse set of interaction techniques to allow the manipulation and analysis of archaeological data and virtual historic artefacts.

## Research Highlights

An interactive, multi-user, immersive archaeological data visualization tool is described.

Survey evaluation elicits insights into where the system provides value and engagement for key stakeholders.

Thematic Analysis provides further design insights for future development of immersive archaeological data visualization tools.
